# Ruptured dermoid cyst of ovary developing into chronic peritonitis; a rare complication diagnosed by contrast CT: A case study

**DOI:** 10.1016/j.amsu.2022.104700

**Published:** 2022-09-15

**Authors:** Kalpana Rai, Binaya Dhakal, Sunil Shahi, Sujit Pant, Suhail Sapkota, Bibek Timilsina

**Affiliations:** a Shree Birendra Hospital, Department of Radiology, Chhauni, Kathmandu, Nepal; b Military Hospital Nepalgunj, Banke, Nepal; c Nepalese Army Institute of Health Sciences- College of Medicine, Nepal

**Keywords:** Dermoid, Chronic peritonitis, Rupture, CT imaging

## Abstract

**Introduction:**

Dermoid cyst also called Mature cystic teratoma is the most common ovarian germ cell tumor of pre-menopausal females, composed of skin, hair, teeth, and sebum covered by thick fibrous tissue. It can present with complications like torsion, rupture, infection, and autoimmune hemolytic anemia. The case highlights the role of imaging in the diagnosis of ruptured dermoid cyst which can have subtle clinical features.

**Case Presentation:**

Herein we present a case of 53 years multiparous postmenopausal female who presented with lower abdominal pain. Examination findings at presentation were normal. 2 years back patient was evaluated for the abdominopelvic mass which was diagnosed radiologically as an ovarian dermoid cyst. This time, Ultrasonography (USG) of the abdomen and pelvis followed by Contrast-enhanced computed tomography (CECT) of the abdomen and pelvis revealed the features consistent with a ruptured dermoid cyst. Exploratory laparotomy and histopathological examination of the specimen confirmed the diagnosis.

**Clinical Discussion:**

Rupture of a dermoid cyst is a very infrequent complication. Following rupture patient may present with peritonitis which may be acute or chronic. Chronic peritonitis may not show any clinically distinguishable features such that the clinical diagnosis of the rupture dermoid cyst is difficult to make. The radiological assessment helps to make an accurate diagnosis so that appropriate surgical intervention can be instituted.

**Conclusion:**

Following the rupture of the dermoid patients may progress to a stage of chronic peritoneal inflammation. At this stage, the radiological assessment may be crucial for appropriate diagnosis and thus further management.

## Highlights

• Dermoid cyst is the most common ovarian germ cell tumor of pre-menopausal females.
• Rupture of an ovarian dermoid cyst is a rare complication.
• Rupture of the dermoid cyst may present with very subtle and marginal symptoms that are not clinically distinguishable.
• The ruptured teratoma can be accurately diagnosed by the contrast CT and hence the appropriate management can be made.

## 1 Introduction and importance

Among the tumors of the germ cell derivatives of the ovary, dermoid/mature cystic teratoma is the most common variant that accounts for 20% of all adult ovarian tumors[1]. [2]. This tumor is classically composed of well-differentiated tissues of all three germ layers that include teeth, hair, skin, fat, sebaceous materials, and bones covered by a thick capsule whose wall may contain calcifications[3]. [4]. [5].10–15% of these tumors are bilateral and are usually incidental findings on medical imaging [5]. It is present most commonly in premenopausal women with the most common age group being under 30 years and the incidence is much lower in postmenopausal women [2]. [5].

Though the tumor is usually asymptomatic and remains an incidental finding, a few times patients present with complications with their corresponding clinical and imaging features [6]. [2]. These complications include torsion (16%), rupture (1–4%), malignant transformation (1–2%), infection (1%) and autoimmune hemolytic anemia (<1%) [3]. [4]. [2]. Sometimes these complications may present in an advanced pregnancy as well [7]. [8].

Herein we present a case of 53 years female with a ruptured dermoid cyst diagnosed with contrast CT. This case report has been reported as per SCARE 2020 criteria [9].

## 2 Case Presentation

History dates back around 2 years to a 53-year-old postmenopausal multiparous woman who presented to gynecological OPD with a complaint of lower abdominal pain. The pain was associated with abdominal heaviness and fullness. It was non-radiating with no known relieving and aggravating factors. Patient had no significant surgical or medical history in the past. There was no relevant family history.

Abdominal examination revealed a cystic/solid globular mass in the lower abdomen with a size of approximately 24 weeks. The mass was smooth, freely mobile with getting below the swelling possible. It was non-tender and non-pulsatile. Examination of the other systems were insignificant.

**Fig. 1:**
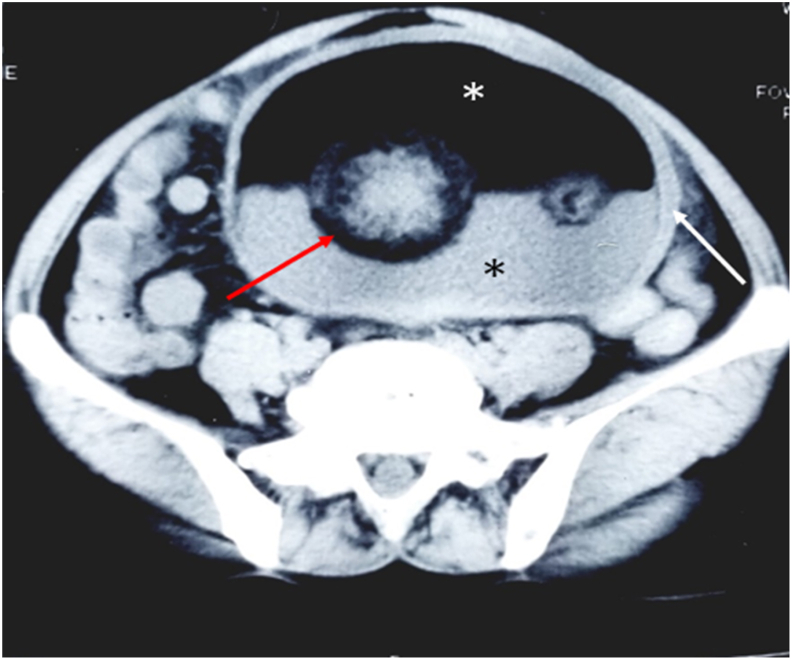
Well-defined large pelvic thick-walled cystic lesion not clearly separable from the ovaries measuring approx. (9.7 × 14.5 × 13.7) cm (white arrow) with fat (white asterisk) fluid (black asterisk) level and a multiple Rokitansky soft tissue nodules (red arrow) at the junction of fat fluid level. (For interpretation of the references to colour in this figure legend, the reader is referred to the Web version of this article.)

Admission was done for further work-up. Ultrasonography (USG) of the abdomen and pelvis done on August 14th^,^ 2020, showed a large well defined, smoothly marginated thick-walled cystic lesion measuring approximately (14.0 × 12.0x10.0) cm in size in the abdominopelvic region with few foci of calcifications within suggestive of a dermoid cyst. It was followed by a Contrast-Enhanced Computed Tomography (CECT) abdomen and pelvis which revealed a large, well-defined, smoothly marginated, predominantly fat attenuating lesion with fat fluid level, few Rokitansky soft tissue nodules, and few calcifications in the abdominopelvic cavity abutting the bilateral adnexal region measuring about (9.7 × 14.5 × 13.7) cm-consistent with dermoid cyst most likely of ovarian origin [Fig. 1]. The tumor marker assessment showed no abnormality (CA-125 -9.5U/ml, CEA-2.8U/ml, and CA19.9–6.4U/ml).

During the hospital stay, the patient contracted COVID-19 infection and was kept in the isolation ward. Apart from mild fever patient did not develop any major symptoms of COVID-19. She was managed with Acetaminophen for fever. Patient denied the hospital stay and wanted self-home isolation. She was discharged on request with the consent signed, explained, and counseled about her disease condition.

2 years later, the patient experienced sudden severe pain and discomfort in her lower abdomen following one and half hours of two-way travel on a motorbike from her home to a nearby town along the unlevelled, graveled off-road in the remote hilly area of Nepal. The pain was sudden, severe, aggravated by movement, and associated with nausea, difficulty breathing, fatigue, chills, and fever. The following day patient visited the local medical shop and took some analgesics, but the pain did not completely resolve. She sought no medical attention in the meantime. However, the symptoms gradually subsided over the next three days except for the dull ache in her lower abdomen.

After 30 days of this event, as the symptoms persisted, the patient presented to the hospital in Gynecological OPD with the complaint of lower abdominal pain. The pain was dull, radiating to her right shoulder with no known relieving or aggravating factors.

This time, on examination, the abdomen was soft, non-tender, and non-distended with no organomegaly or palpable mass.

**Fig. 2:**
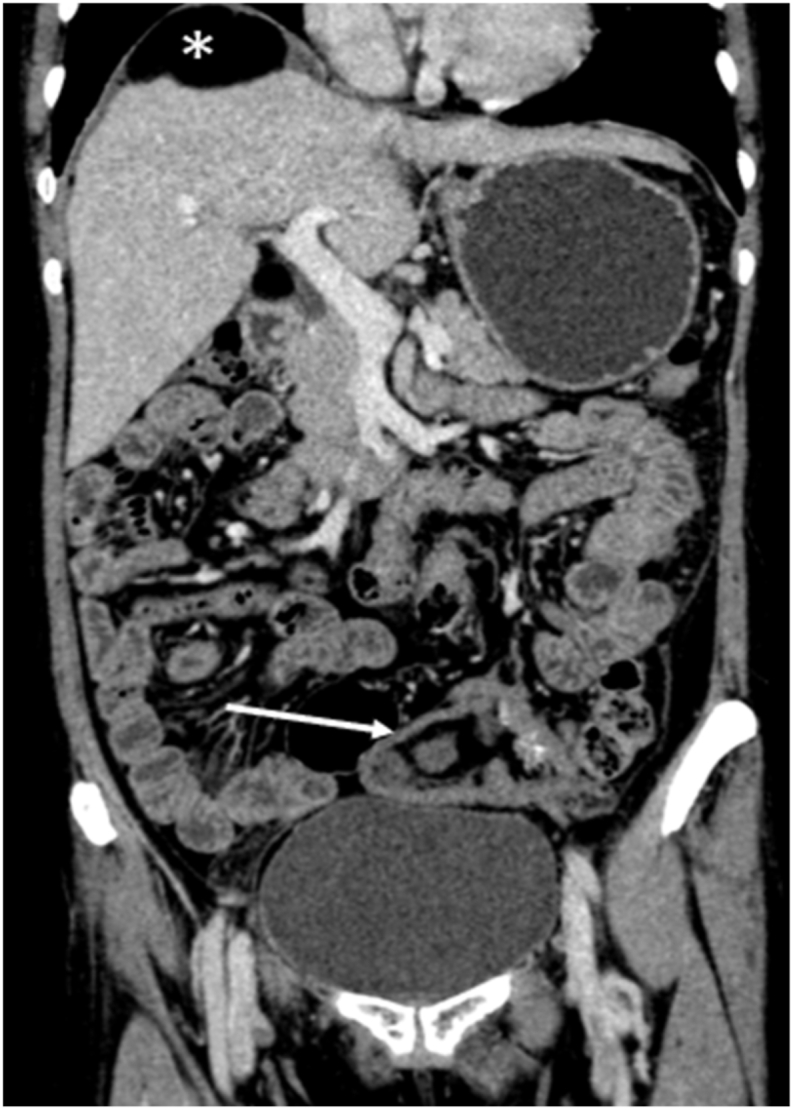
Well-defined lobulated thick-walled left adnexal cystic lesion not clearly separable from the ovaries measuring approx. (6.3 × 8.5 × 4.6) cm with multiple calcific foci within (white arrow) and associated perilesional fat stranding along with fat attenuating lesion in right subdiaphragmatic region (white asterisk).

Her blood investigations showed no derangements. USG done on 31^st^ May 2022 showed a lobulated thick-walled left complex adnexal cystic lesion. It was followed by CECT abdomen and pelvis the next day which revealed a significant decrease in size of the previously described lesion with lobulations in the wall suggesting rupture. The lesion measured approx. (6.3 × 8.5 × 4.6) cm with multiple calcific foci, thick strands, and areas of fat attenuation within. Multiple fat attenuating lesions were seen in different intra-peritoneal spaces including subdiaphragmatic, subhepatic, perisplenic, and perilesional regions. [Fig. 2].

**Fig. 3:**
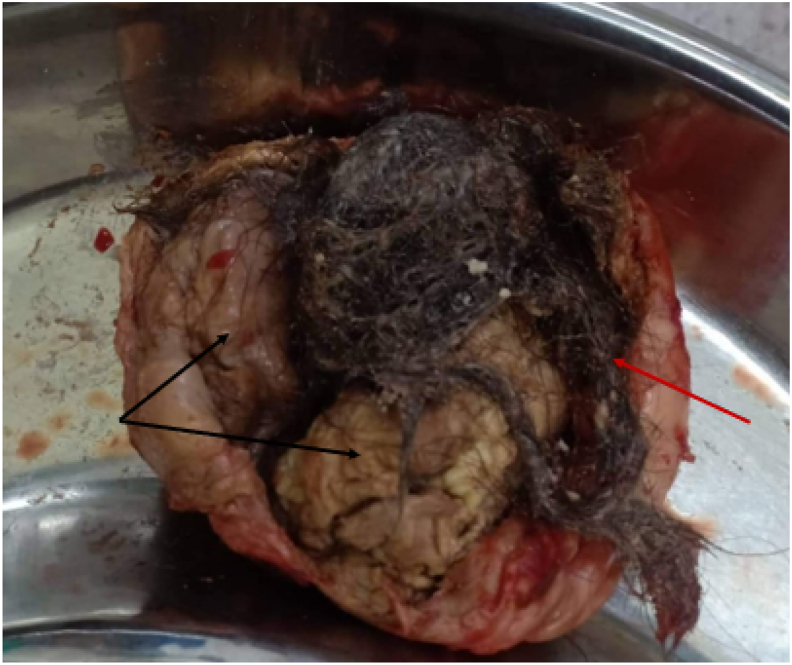
Gross picture of the ruptured dermoid cyst post-surgery showing hair particles (red arrow), caseous materials and fat contents (black arrow). (For interpretation of the references to colour in this figure legend, the reader is referred to the Web version of this article.)

With the imaging diagnosis of a ruptured dermoid cyst, the patient was admitted to the hospital. On 6^th^ June 2022 patient underwent exploratory laparotomy which revealed a ruptured dermoid cyst of approximately (9.0 × 8.5x4.5) cm with a rent of (2.0 × 2.0) cm from where the caseous material along with hair follicles was seen coming out [Fig. 3]. The peritoneal cavity showed the signs of chronic inflammation. The cyst was adherent to the adjacent bowel loops from all sides. A serosal tear was evident in the sigmoid colon. She was managed with dermoid removal, left oophorectomy, and serosal repair of the sigmoid colon. The postoperative period was uneventful, and the patient was discharged on her 5th postoperative day. She made a good recovery with no significant complaints in her follow-up postoperatively. The histopathological examination revealed a ruptured dermoid cyst with a giant cell reaction consistent with chronic granulomatous inflammation with no evidence of malignancy.

## 3 Clinical discussion

A Dermoid is a slow-growing benign tumor of the ovary that usually remains clinically silent, and is incidentally detected during medical imaging [2]. [5]. The main complications of this tumor include torsion (16%), rupture (1–4%), malignant transformation (1–2%), infection (1%), and autoimmune hemolytic anemia (<1%) [3]. [4]. [2]. Because of the thick capsule of the dermoid cyst, spontaneous rupture is a very rare complication [10]**.** Although the precise etiology of the rupture of a dermoid cyst is largely unknown, some of the possible etiologies that are explained in literature are infection, infarction or malignant change of the tumor, direct trauma, pregnancy changes, and rapid cyst expansion [11].

After the rupture of the dermoid cyst, the release of tumor contents can cause peritonitis. The presentation can be acute or chronic [1]. [2]. The latter is more common [1]which is the same as in our case here. Acute peritonitis presents with features of the acute abdomen or shock [1]. Chronic peritonitis results from a tiny break in the cyst wall which thereby releases cyst content causing granulomatous inflammation of the peritoneum [1]. [2]. [5]. Such patients with chronic granulomatous peritonitis secondary to rupture of the dermoid cyst may present with very subtle and marginal symptoms that are not clinically distinguishable [1]. [2]. Also, there are few reported cases with no remarkable clinical features following the rupture of the dermoid [12]. Hence imaging modalities specifically CECT imaging can make an accurate diagnosis of the ruptured dermoid [3]. [1]. [2]. [5]. [13]. This is achieved by noting the discontinuity in the wall of the dermoid during the imaging [1]. [2]. [5]. [10]. Since ruptured dermoid causes spillage and deposition of fat within the peritoneal cavity, the CT imaging has very high sensitivity and specificity to demonstrate the fat attenuating lesions along the peritoneum and intraperitoneal organs and also a characteristic hypoattenuating fat-fluid levels in the ante dependent pockets within the peritoneal cavity, typically below the right hemidiaphragm, a pathognomonic finding [10].

Here in our case, it is likely that the dermoid got ruptured with a small rent when the patient traveled on a motorbike along the unlabeled off-road. The ruptured contents disseminated in the abdominal cavity causing peritonitis which was initially acute for the first three days that subsequently developed into chronic granulomatous inflammation of the peritoneum due to continuous leakage from the rent. By the time the patient developed chronic peritonitis the symptoms were very vague, subtle, and clinically indistinguishable from other causes of abdominal pain. In this situation, the USG and CECT were key to the diagnosis with their characteristic findings as mentioned above. It is also notable that the fat deposition in the right subdiaphragmatic region which was evident in the CECT caused the patient to have right shoulder pain which is explained by the diaphragmatic irritation by the fat. The rupture with the rent was later confirmed in the surgery which also showed chronic inflammatory changes along with fat deposition within the peritoneal cavity.

## 4 Conclusion

Dermoid can often present with an uncommon complication like a rupture. It might be a spontaneous rupture or due to any inciting trauma. Following the rupture, the patient can develop acute or chronic granulomatous peritonitis. Patients who develop chronic granulomatous peritonitis may not be clinically evident and imaging can be diagnostic with its characteristic finding. Specifically, contrast CT has very high sensitivity and specificity to diagnose such cases. It is therefore advisable to perform the imaging in the patients who present with vague abdominal pain inconsistent with acute peritonitis and have a well-known history of mature cystic teratoma to rule out possible rupture of the teratoma. The ruptured teratoma can be accurately diagnosed via the imaging modalities and hence the appropriate management can be intervened.

## Source of funding

None.

## Research registration number

Not applicable.

## Provenance and peer review

Not commissioned, externally peer reviewed.

## Ethical approval

N/A.

## Please state any sources of funding for your research

None.

## Author contribution

Author 1: Led data collection, concept of study, contributed in writing the case information.

Author 2: Literature review, writing initial draft, revising, and editing the manuscript.

Author 3: Literature review and writing case information.

Author 4: Literature review, revising and editing the manuscript.

Author 5: Revised and edited the rough draft into final manuscript.

Author 6: Revised and edited the rough draft into final manuscript.

All authors were involved in manuscript drafting and revising and approved the final version.

## Registration of research studies

1. Name of the registry: N/A.
2. Unique Identifying number or registration ID: N/A.
3. Hyperlink to your specific registration (must be publicly accessible and will be checked): N/A.

## Guarantor

Binaya Dhakal, Military Hospital Nepalgunj, Banke, Nepal. Email: dhbinay@gmail.com Phone: +977–9849764844.

## Consent

Written informed consent was obtained from the patient for publication of this case report and accompanying images. A copy of the written consent is available for review by the Editor-in-Chief of this journal on request.

## Declaration of competing interest

None.
